# Asymptomatic *Plasmodium vivax* infections among Duffy-negative population in Kedougou, Senegal

**DOI:** 10.1186/s41182-018-0128-3

**Published:** 2018-12-29

**Authors:** Makhtar Niang, Rokhaya Sane, Abdourahmane Sow, Bacary D. Sadio, Sophy Chy, Eric Legrand, Ousmane Faye, Mawlouth Diallo, Amadou A. Sall, Didier Menard, Aissatou Toure-Balde

**Affiliations:** 1 Immunology Unit, Pasteur Institute of Dakar, BP 220 Dakar, Senegal; 2 Department of Animal Biology, Cheikh Anta Diop University of Dakar, Dakar, Senegal; 3 Arbovirus and Viral Hemorrhagic Fevers Unit, Pasteur Institute of Dakar, BP 220 Dakar, Senegal; 4 West African Health Organization, Ouagadougou, Burkina Faso; 5 Malaria Molecular Epidemiology Unit, Institute Pasteur in Cambodia, Phnom Penh, Cambodia; 6 Groupe Génétique du Paludisme et Résistance, Unité Biologie des Interactions Hôte-Parasite, Institut Pasteur, Paris, France; 7 Medical Entomology Unit, Pasteur Institute of Dakar, BP 220 Dakar, Senegal

**Keywords:** Malaria, *Plasmodium vivax*, Asymptomatic carriage, Duffy-negative, Children

## Abstract

**Background:**

In the southeastern Senegal, the report of *Plasmodium vivax* infections among febrile patients in Kedougou constitutes a new emerging health problem.

**Methods:**

Samples from 48 asymptomatic schoolchildren sampled twice a year over 2 years were used to explore the reservoir of *P. vivax* parasite infections in this region. Both Duffy genotyping and *Plasmodium* species diagnostic assays were performed.

**Results:**

PCR assays detected *Plasmodium* genomic DNA in 38.5% (74/192) of samples. Pure *P. falciparum* and *P. vivax* infections were identified in 79.7% (59/74) and 20.3% (15/74) of samples, respectively. All schoolchildren were classified as Duffy-negative by genotyping. *P. vivax* infections were detected in five children: in two children during both years, in one child in 2010 and on May 2011, and only in 2010 for the remaining two children.

**Conclusions:**

This unexpectedly high proportion of *P. vivax* infections in asymptomatic Duffy-negative children highlights to consider vivax malaria as an emerging problem in Senegal.

## Background

Malaria remains a main global cause of death from parasitic diseases threatening approximately half of the world’s population and causing debilitating illness in more than half a million people [1]. Although *Plasmodium falciparum* remains the deadliest human-infecting *Plasmodium* species in Africa [1], *P. vivax* is geographically the most widely distributed malaria parasite, and is yearly responsible of 80–300 million clinical cases, and up to 2.5 billion people are globally at risk [2]. *P. vivax* has been for long believed to be almost completely absent in large parts of sub-Saharan Africa [3] due to the high prevalence of the Duffy-negative phenotype which is supposed to confer a complete protection against *P. vivax* malaria [4].

However, this widely accepted dogma has been recently challenged by increasing reports of *P. vivax* infections in Duffy-negative individuals [5–9], especially in West and Central Africa where previously *P. vivax* infections were not detected by the conventional microscopy and rapid diagnostic tests [6, 7]. The increasing use of sensitive molecular diagnostics has as expected confirmed high incidences of *P. falciparum* infections in Africa, but also revealed instances of *P. vivax* infections in many African countries [5, 7, 10–12].

Vivax malaria is considered to be a chronic infection with a typically mild clinical course [13], due to unique biological features of the parasite including dormant liver-stages relapsing weeks, months, or years after the initial infection, development in mosquito vectors at lower ambient temperatures, and low parasite density. Accordingly, *P. vivax* infections contribute significantly to the malaria parasite reservoir and potentially serve as a significant source of transmission that could impair malaria control and elimination efforts.

Following our recent reports of *P. vivax* infections among febrile patients [11] and asymptomatic schoolchildren in Kedougou region, southeastern Senegal [14], this study was undertaken to survey molecular signatures of true *P. vivax* infections in the same cohort of asymptomatic schoolchildren.

## Methods

The samples used in this study were collected from Kedougou region, southeastern Senegal as part of a project investigating arboviruses infections. Details of Kedougou region including malaria indicators, population, climate, rainfall, landscape, and fauna have been previously reported [11, 15]. We tested in total 192 samples collected from 48 asymptomatic schoolchildren (C1 to C48) who were followed during two consecutive years (2010 and 2011), and sampled twice each year (May and November) corresponding to the dry and rainy seasons, respectively. For this study, sera samples were withdrawn from a collection of archived biological specimens established as part of a project investigating asymptomatic and symptomatic arboviruses infections.

The study was examined and approved by the Senegalese National Health Research Committee under the reference 0081MSP/DS/CNRS. A healthcare worker was trained to conduct interviews to explain the study objectives, benefits, and risks to parents/guardians and school administrators before inclusion. Written informed consents were obtained from parents/guardians of children participants.

For this study, samples were tested for *Plasmodium* spp. infections with two well-validated approaches using genomic DNA (gDNA) extracted from frozen serum samples as previously described [16, 17]. First, the presence of *Plasmodium* genus and species DNA was tested by nested PCR targeting the *18S ssrRNA* genes as previously described [18]. The *Plasmodium* genus-specific and species-specific PCR amplifications were performed as detailed previously [11]. Second, blinded DNA samples of the entire cohort were sent to an independent laboratory in Cambodia for confirmation of the results using a real-time screening and four species identification PCR assays targeting the *Plasmodium cytochrome b* gene as described previously by Canier et al. [19].

Sequences to detect SNP in the GATA-1 transcription factor binding site at nucleotide position − 33 (t, wild-type; c, erythrocyte silent) were obtained to determine the Duffy genotype of all individuals, as previously described [5]. PCR products were sent to Macrogen (Seoul, South Korea) for Sanger sequencing. The electropherograms were analyzed on both strands with CEQ2000 Genetic Analysis System software (Beckman Coulter, Brea, CA, USA). Nucleotide sequences were compared to the glycoprotein Duffy group antigen sequence (GenBank accession No. S76830).

## Results

At the time of the first sampling (May 2010), children were aged 8 to 11 years old with a mean age of 9. The sex ratio M/F was 1.4 (28/20). All children were asymptomatic (axillary temperature < 37.5 °C) over the four sampling periods.

Of the 192 samples tested (4 × 48), 38.5% (74/192) were found positive by using the genus-specific *18S ssRNA* PCR assay (Table 1). The proportions of *Plasmodium*-positive samples were significantly higher in November (58.3% in 2010 and 43.7% in 2011) compared to May (31.2% in 2010 and 20.8% in 2011, *p* = 0.007 and *p* = 0.01, respectively, Fisher’s exact test) for both years. As expected, species-specific nested PCR assay revealed *P. falciparum* accounted for the majority of infections and was present as pure infection in 79.7% (59/74) of *Plasmodium-*positive samples. No single or mixed *P. malariae* or *P. ovale* infections were detected among the screened samples. We identified additionally 15 pure *P. vivax*-positive samples from 5 children. The proportions of *P. falciparum* and *P. vivax* infections were respectively 20.8% (10/48) and 10.4% (5/48) in May 2010; 47.9% (23/48) and 10.4% (5/48) in November 2010; 14.6% (7/48) and 6.2% (3/48) in May 2011 and 39.6% (19/48) and 4.2% (2/48) in November 2011 (Table 1). All PCR data were confirmed later by *Plasmodium cytochrome b* gene-based real-time PCR.

**Table 1:** Proportion of asymptomatic *Plasmodium* infections detected among 48 schoolchildren over 2 years sampling period in Kedougou, Senegal

Sampling period	Plasmodium genus screening		Total	Malaria species screening*		Total
	Positive *n* (%)	Negative *n* (%)		*P. falciparum* *n* (%)	*P. vivax* *n* (%)	
May 2010	15 (31.2)	33 (68.75)	48	10 (66.7)	5 (33.3)	15
November 2010	28 (58.3)	20 (41.66)	48	23 (82.1)	5 (17.9)	28
May 2011	10 (20.8)	38 (79.17)	48	7 (70.0)	3 (30.0)	10
November 2011	21 (43.7)	27 (56.25)	48	19 (90.5)	2 (9.5)	21
Total	74 (38.5)	118 (61.5)	192	59 (79.7)	15 (20.3)	74

*Number and percentage were expressed relative to *Plasmodium*-positive samples

**Fig. 1:**
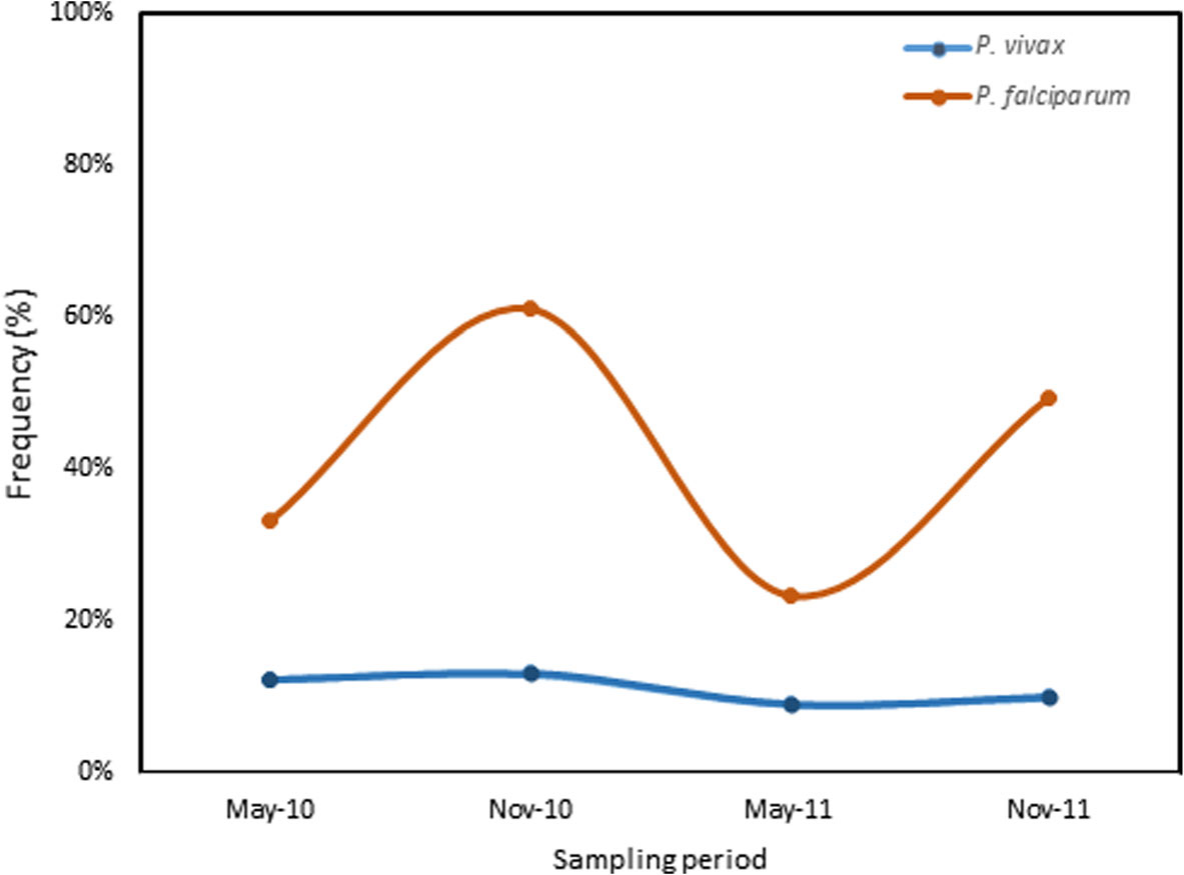
Distribution of positive *P. falciparum* and *P. vivax* infections in the cohort during follow-up

The proportions of positive *P. vivax* samples did not vary significantly during follow-ups and were 12.5%, 13.2%, 9.1%, and 10% respectively on May 2010, November 2010, May 2011, and November 2011 (*p* = 0.28, chi-squared test, Fig. 1). By contrast, proportions of positive *P. falciparum* increased slightly from May to November: from 20.8% to 47.9% in 2010 (*p* = 0.009, Fischer’s exact test) and from 14.5% to 39.5% in 2011 (*p* = 0.01, Fisher’s exact test, Fig. 1).

Details regarding the age and sex of the five *P. vivax*-infected children (identified as C6, C8, C13, C29, and C47) and the dynamics of individual *P. vivax* carriage across the sampling periods are provided in Table 2. *P. vivax* parasites were present in all children in May and November 2010, in three children (C8, C13, and C29) in May 2011 and in two children (C8 and C29) in November 2011 (Table 2). All schoolchildren were classified as Duffy negative (FY*B^ES^/*B^ES^) including the five *P. vivax-*infected ones.

**Table 2:**
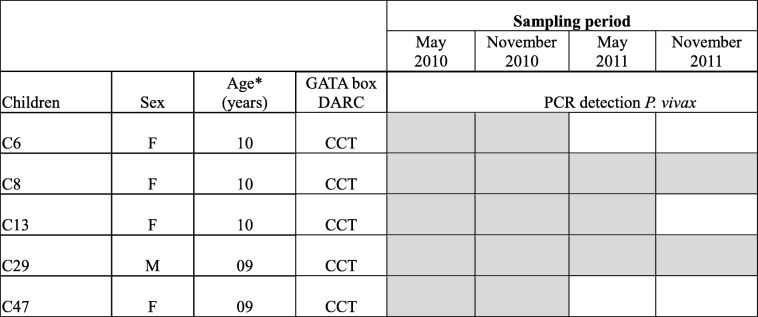
Dynamic carriage of *Plasmodium vivax* parasites among the five Duffy-negative children across sampling periods (2010–2011), Kedougou, Senegal

*Age at inclusion; gray and white boxes represent the presence and absence of *P. vivax*, respectively

## Discussion

A new vision of *P. vivax* transmission in Africa has recently emerged with increasing evidence of the parasite’s presence in Duffy-negative individuals [2]. Previous reports, including ours, strongly challenge the paradigm that Duffy-negativity confers a full protection against *P. vivax* infection and suggest that *P. vivax* infections in symptomatic subjects probably represent only the tip of the iceberg [7, 14, 20, 21]. Accordingly, survey of *P. vivax* infections in asymptomatic individuals as in the present study, would provide better estimates of the silent *P. vivax* parasites reservoir in a given community or setting.

We herein confirm an unexpectedly high proportion (20.3%) of *P. vivax* infections among asymptomatic schoolchildren in Kedougou region by using molecular detection of *P. vivax* DNA although this approach cannot firmly confirm the presence of active erythrocytic infections. The possibility of our PCR technique picking up DNA from pre-erythrocytic infections of *P. vivax* as shown by Abkallo et al. (2015). However, these findings are in line with our previous report detecting 53% positive IgG antibody responses to *P. vivax* MSP1 antigen with the same samples [15]. In our cohort, *P. vivax* infections in children C8 and C29 over a period of 2 years might be due to persistent *P. vivax* parasites. This hypothesis could be supported by the similar proportions of *P. vivax* infections across sampling periods. Nevertheless, the possibility of relapses or re-infection during follow-up cannot be ruled out. The failure to detect *P. vivax* parasites in May and November 2011 in children C6 and C47 and in November 2011 in child C13 might be due to the clearance of the parasites from the peripheral blood or a strong reduction of the parasite load at density below the detection limit of our diagnostic methods. In addition, the failure to detect both *P. malariae* and *P. ovale* in this study was somehow surprising since recent studies have reported the presence of both species in the area [11, 22].

Finally, the current findings of an important asymptomatic *P. vivax* reservoir along with the recent report of *P. vivax* infections among febrile patients in Kedougou region [11] suggest that *P. vivax* parasites circulate in this area where Duffy negativity is fixed. The growing evidence of *P. vivax* infections in Kedougou region where transmission of the major malaria parasite species *P. falciparum* is still active has thus brought in a new paradigm for the control program that might require immediate attention, in order to sustain the limited success gained to date concerning malaria control in the area. Further research is needed to better document the reservoir of *P. vivax* infections in this population and its relevance to malaria transmission and clinical malaria incidence.

## Conclusions

The study reveals an unexpectedly high proportion of *P. vivax* infections among asymptomatic Duffy-negative children in the Kedougou region. This points out critical needs of further studies investigating factors that drive *P. vivax* transmission in this particular area where control efforts towards *P. falciparum* have shown limited impact. We do not know if vivax malaria cases were missed previously or if we are facing to the emergence of strains using alternative Duffy-independent invasion pathways that may lead to an expansion of *P. vivax* malaria into Duffy-negative populations. Whatever the reason, this is a public health concern which demands attention.

## Abbreviations

DARC: Duffy antigen/chemokine receptor
DNA: Deoxyribonucleic acid
gDNA: Genomic DNA
*P. falciparum*: *Plasmodium falciparum*
*P. vivax*: *Plasmodium vivax*
PCR: Polymerase chain reaction
ssRNA: Small subunit ribonucleic acid
